# Supplementary material for: Photonic Weyl degeneracies in magnetized plasma
Source: Nat Commun. 2016 Aug 10;7:12435. doi: 10.1038/ncomms12435 (PMC4987518; doi:10.1038/ncomms12435)
Supplement: Supplementary Information — Supplementary Figures 1-13, Supplementary Notes 1-10 and Supplementary References [file ncomms12435-s1.pdf]

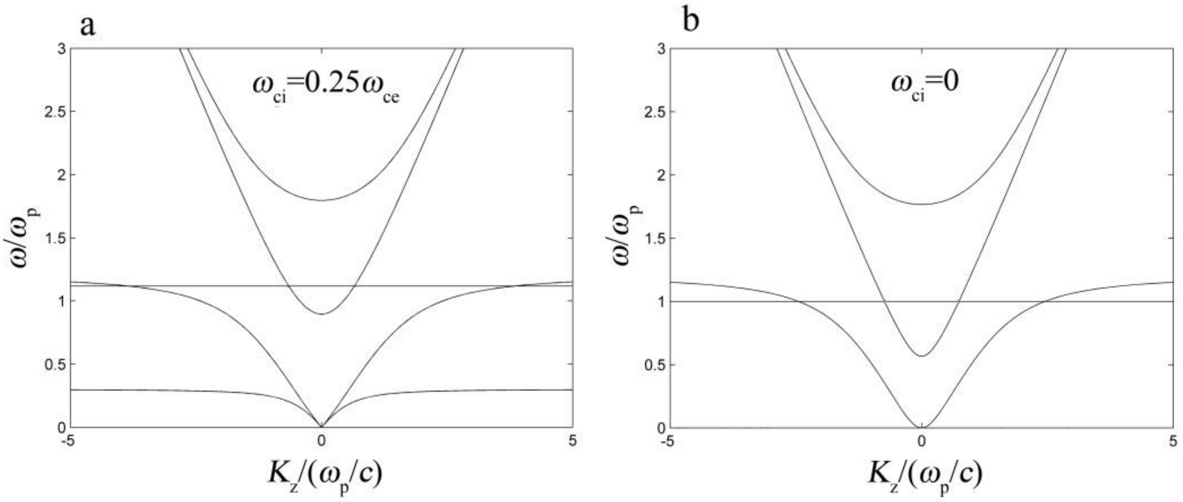

Supplementary Figure 1 | Band structures with/without exaggerated ω_{pi} and ω_{cp}. (a) Band structure with $\omega_{ce} = 1.2\omega_{pe}$ and $\omega_{ci} = 0.25\omega_{ce}$ gives an extra low energy band. (b) Omitting the ions leaves high energy behaviours almost intact. Hamiltonian with both ions and electrons considered is give in Supplementary Note 1.

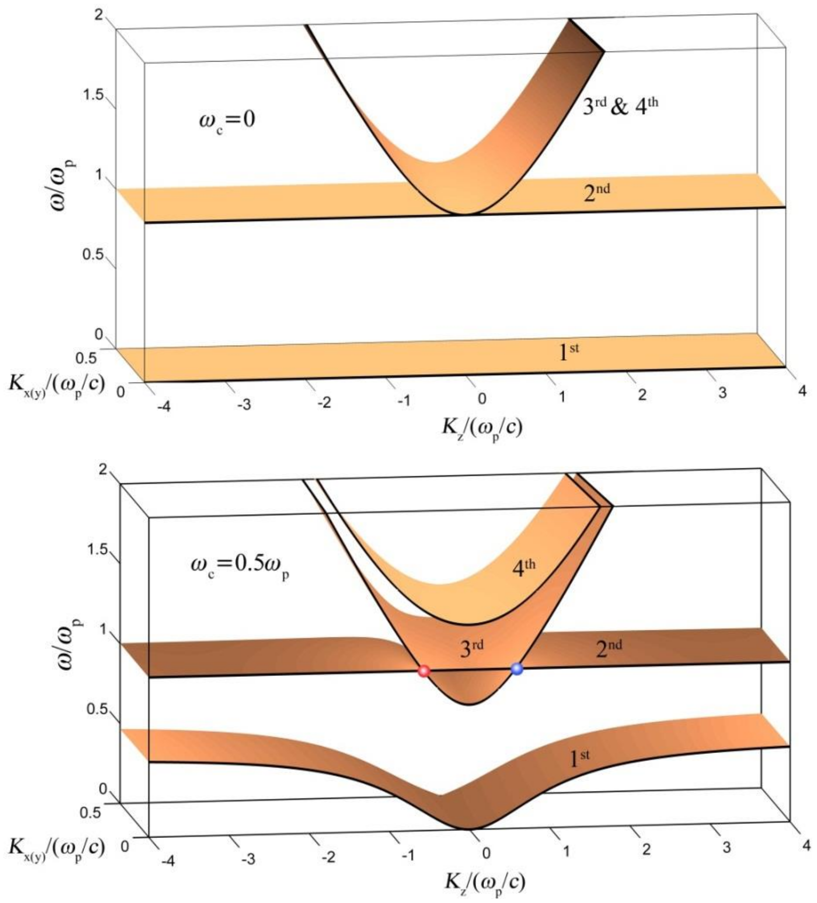

Supplementary Figure 2 | Band structures with different value of ω_c .(a) When $\omega_c = 0$, the magnetized plasma is simply a lossless 3 dimensional metal. The first band become a flat band in zero frequency. The second band is the longitudinal mode flat band in plasma frequency. The third and fourth band are two degenerate bands on higher frequency. (b) When $\omega_c = 0.5\omega_p (B = 0\ T,\ 0.16\ T)$, degeneracy between third and fourth bands are lifted, and the longitudinal mode flat band reduces to a straight line along k_z axis. As is shown in Fig. 1b in the main text, only inner Weyl points are at present. They are marked as red/blue spots in the picture.

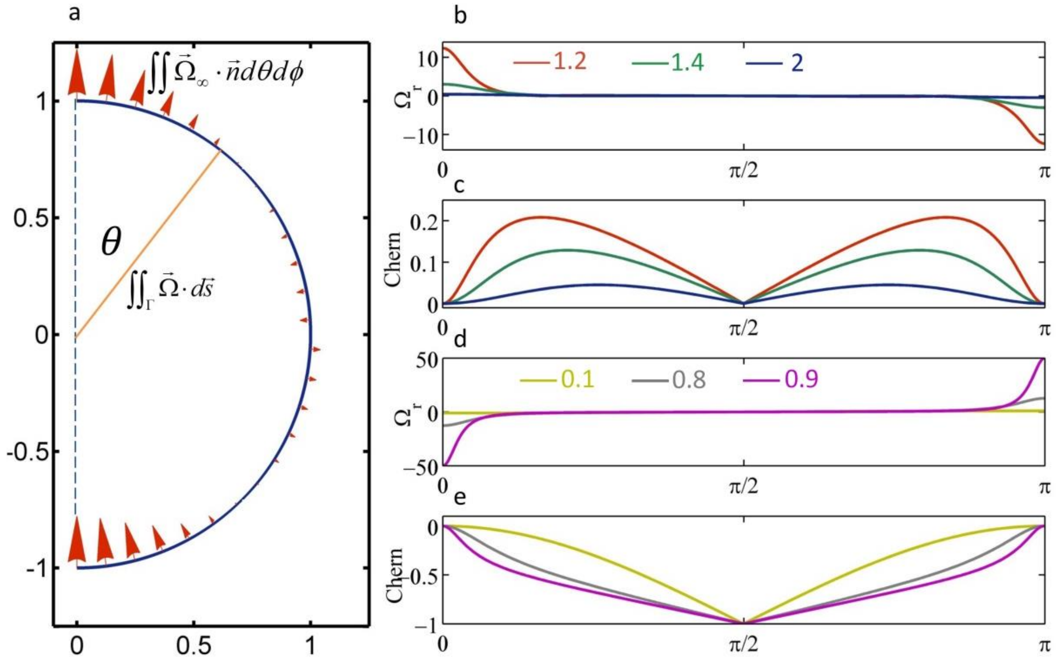

Supplementary Figure 3 | Infinity Hamiltonian's Berry curvature. (a) Berry curvatures in infinity projected onto a unit sphere when $\omega_c = 1.4\omega_p$. The orange line represents the hyperbolic EFSs and θ is the angle between the asymptote of hyperbolic EFS and the z axis. Berry curvatures (b, d) and Chern numbers (c e) are plotted as functions of θ under different ω_c. A noticeable difference is observed between $\omega_c < \omega_p$ and $\omega_c > \omega_p$.

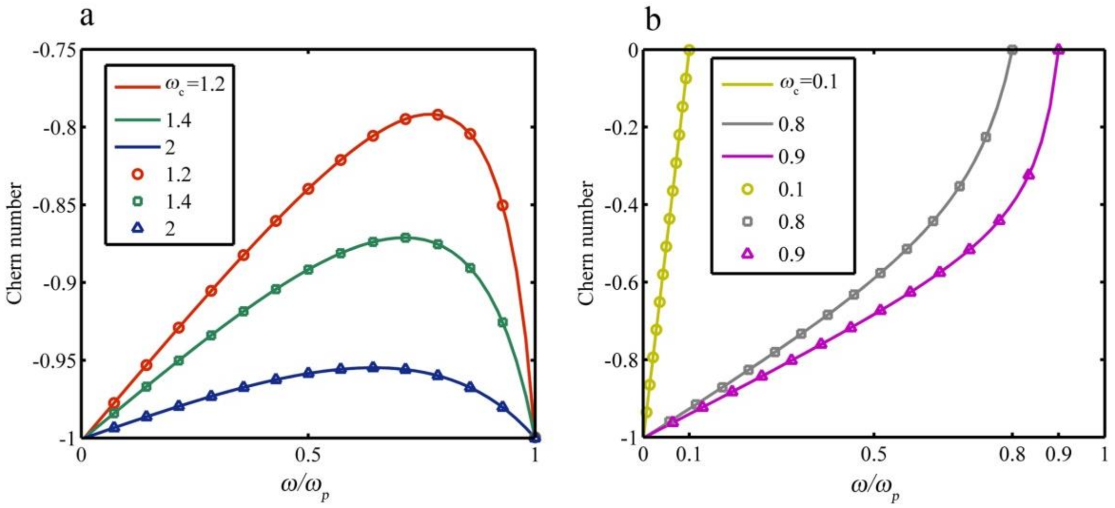

Supplementary Figure 4 | Chern number of Hyperbolic EFSs. Chern number computed by the left hand side (coloured dots) and the right hand side (coloured lines) of Supplementary Equation 1 exactly match each other for various frequencies and ω_c.Results for $\omega_c < \omega_p$ are shown in (a) and $\omega_c > \omega_p$ are in (b). In (b), when ω_c is smaller than ω_p, frequency is only evaluated in the range: [0, ω_c], since hyperbolic EFS is only at presence in this frequency range.

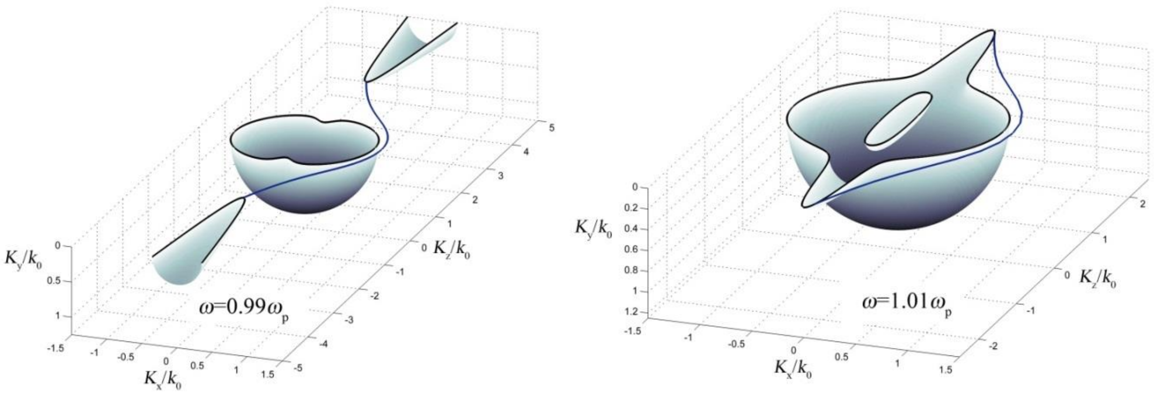

Supplementary Figure 5 | 'Fermi arcs' at shifted frequencies. Even though the integral of Berry curvatures are generally not quantized, 'Fermi arcs' still keep connecting. Spatial dispersions of 'Fermi arc' between magnetized plasma and vacuum at (a) $\omega = 1.01\omega_p$ and (b) $0.99\omega_p$ are shown. they keep connecting even when the EFSs collapse into closed surfaces whose Chern number vanishes.

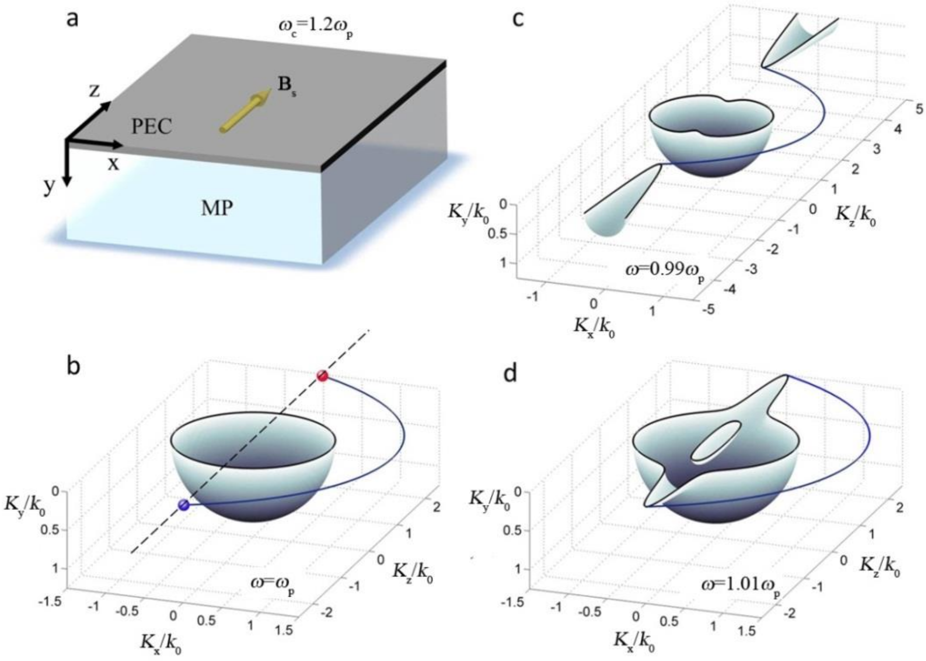

Supplementary Figure 6 | 'Fermi arcs' between magnetized plasma and PEC. 'Fermi arcs' could also be found when magnetized plasma is attached to PEC. (a) Schematic diagram of the scheme for realizing 'Fermi arcs'. (b) At the 'Weyl degeneracy' frequency, there is a 'Fermi arc' connecting the two Weyl points with opposite chirality (marked as red and blue respectively). (c-d)Away from the Weyl points and the EFS in middle, there is a longitudinal plasma mode (marked as dashed line) along the z axis. The 'Fermi arc' remains present at shifted frequency equals $0.99\omega_p$(c). When $\omega = 1.01\omega_p$(d), there are no open EFSs and total Chern number for the closed EFSs are strictly zero, however the 'Fermi arc' still remains present.

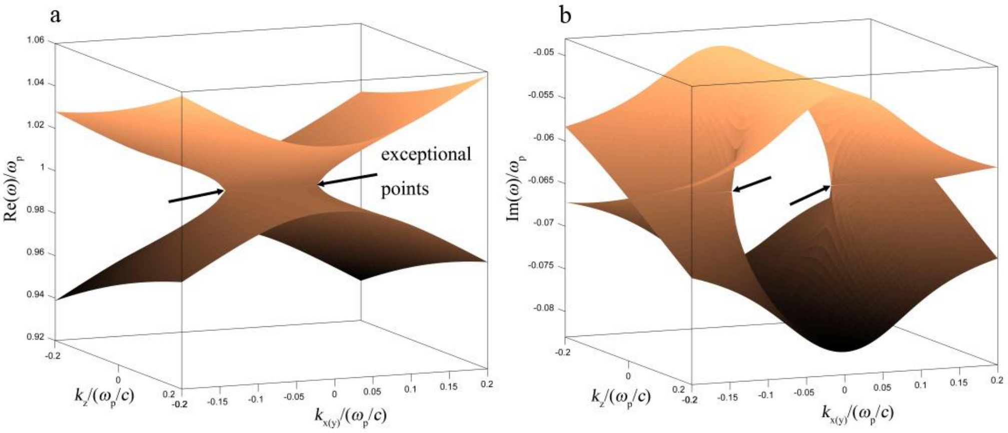

Supplementary Figure 7 | Plasmon Weyl points under loss. Real(a) and imaginary(b) parts of eigen value of Weyl point under loss. The two exceptional points are indicated by the black arrows. Topological feature of this non-hermitian model is discussed in Supplementary Note 6.

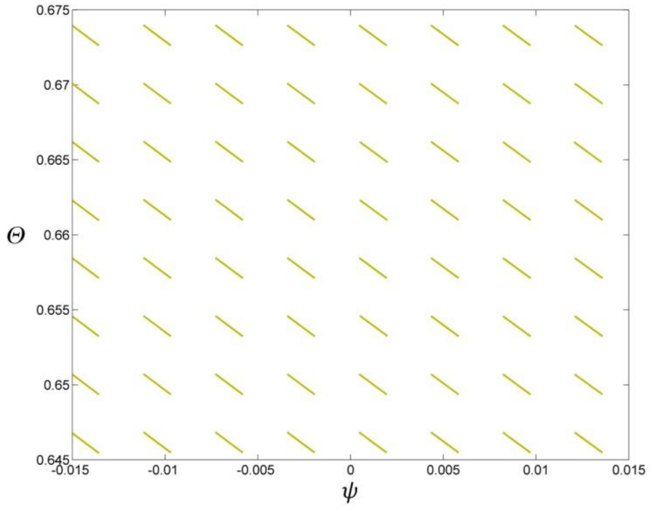

Supplementary Figure 8 | Polarization of the background mode. It agrees with the above analytical expression (Supplementary Note 9)that their projection to the interface are linearly polarized. Moreover, it is apparent that variation of the background field near the plasmon 'Weyl point' is negligible.

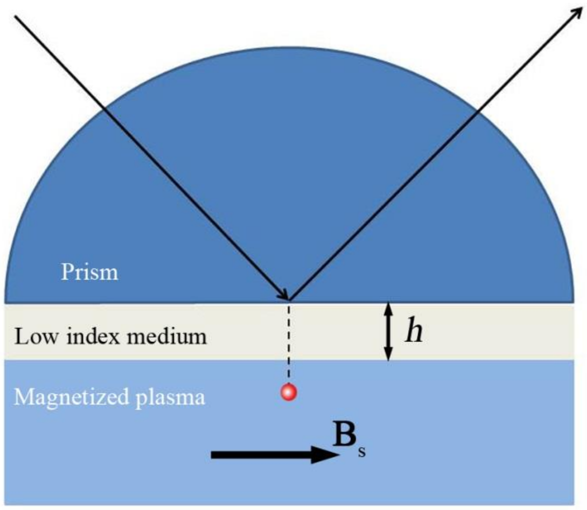

Supplementary Figure 9 | Spherical prism configuration to detect polarization e.igen-states around Weyl point. A low index medium layer with thickness h can be added to demonstrate the polarization features introduced by 'Fermi arc'. See Supplementary Note 10 for detailed discussion

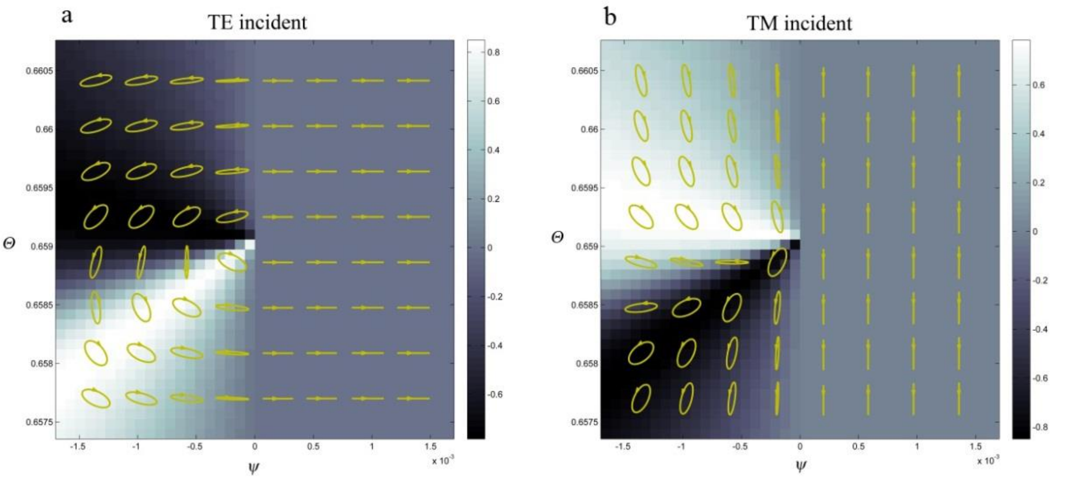

Supplementary Figure 10 | Half plane chirality of different polarized incident EM wave around negative Weyl point. The polarization feature for TE(a) and TM(b) incident only happens in the negative k_x half plane

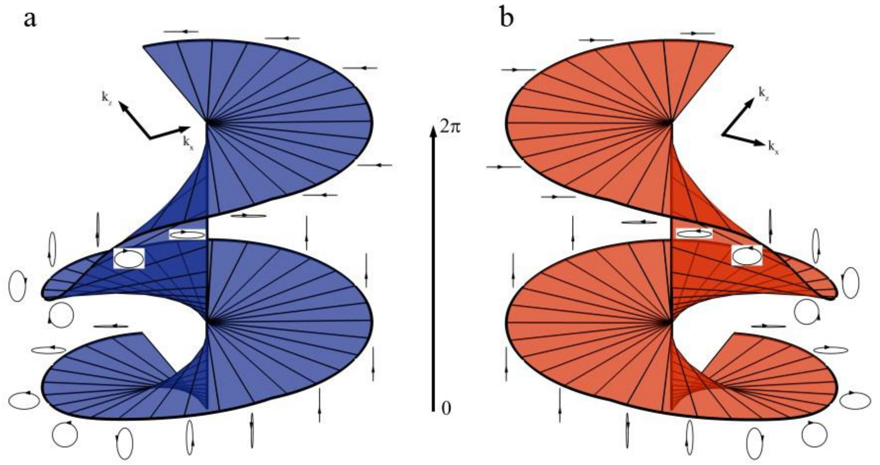

Supplementary Figure 11 | Phase of eigen-polarizations around Weyl points with opposite chirality. It is right handedness vortex for negative Weyl point(a), while left handedness vortex for positive ones(b).

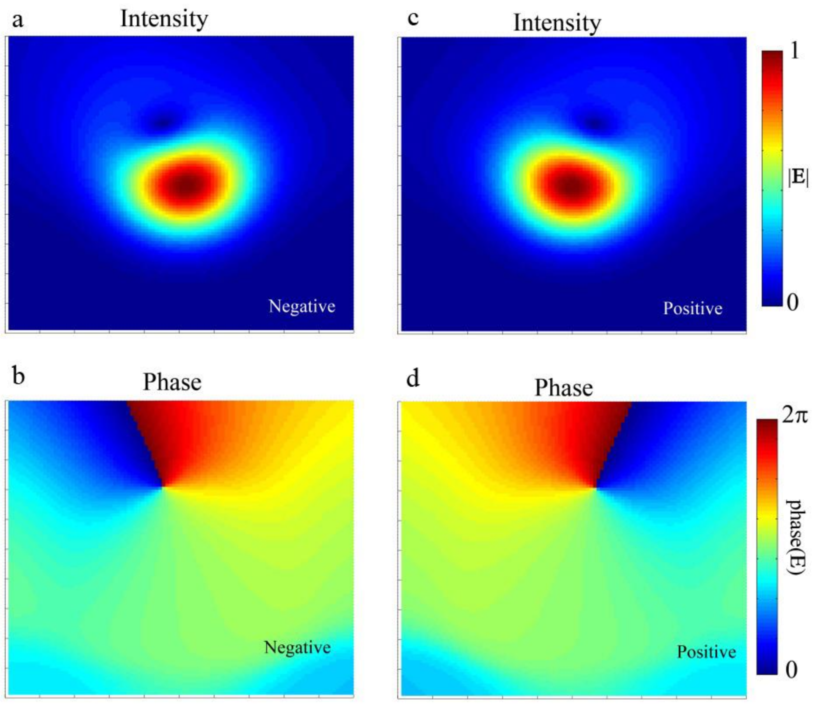

Supplementary Figure 12 | Intensity and phase profile of reflected TM-polarized Gaussian beam The beam waist is 20 times of free space wavelength ensure only the momentums near to Weyl point are included incident on negative(left column) and positive (right column) Weyl points. The dark point in intensity is a phase vortex with optical angular momentum. For opposite-chirality Weyl points, topological charge of the vortex are ± 1, consistent with analysis of phase vortex of polarization eigen-states.

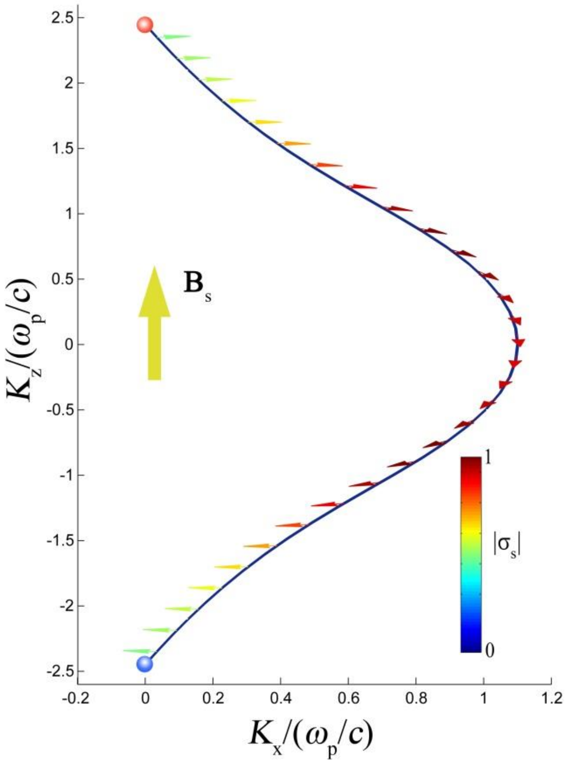

Supplementary Figure 13 | Spin texture on 'Fermi arc' connecting Weyl points. Different Weyl points gives opposite value of $\mathbf{k}\cdot\boldsymbol{\sigma}_s$, detailed in Supplementary Note 11.

Supplementary Note 1. Derivation of magnetized plasma's Hamiltonian formalism

The classical equations of motion of a free electron gas under a static magnetic field can be written as:

$$\frac{d^2\mathbf{P}}{dt^2} - \mathbf{B}_s \times \frac{d}{dt}\mathbf{P} + \Gamma\frac{d}{dt}\mathbf{P} = \omega_p{}^2\mathbf{E}$$

Where ω_p is plasma frequency, $\mathbf{B}_s = [0,0,\omega_\mathrm{c}]$ refers to the static bias magnetic field, and ω_c is cyclotron frequency. ω_p and ω_c can be tuned by plasma density and strength of static bias magnetic field respectively. In this letter, we consider realistic plasma density equals $1 \times 10^{12} cm^{-3}$ that corresponds to $\omega_\mathrm{p} = 5.64 \times 10^{10} rad\, s^{-1}$. ω_c is within range $\{0 < \omega_\mathrm{c} < 1.2\omega_\mathrm{p}\}$ and corresponds to strength of static magnetic field $0T < |\mathbf{B}_\mathrm{s}| < 0.385\, T$, which is well within experimental realizable regime. Next we define:

$$\frac{d}{dt}\mathbf{P} = \mathbf{V}$$

$\mathbf{P}$ and $\mathbf{V}$ are, respectively, the polarization field and electron's velocity field. By coupling the equations of motion with Maxwell equations, we have:

$$\begin{bmatrix} 0 & -c\mathbf{K}\times & -iI \\ c\mathbf{K}\times & 0 & 0 \\ i\omega_p{}^2\mathrm{I} & 0 & \omega_c\Delta \end{bmatrix}\begin{bmatrix}\mathbf{E}\\ \mathbf{H}\\ \mathbf{V}\end{bmatrix} = \omega\begin{bmatrix}\mathbf{E}\\ \mathbf{H}\\ \mathbf{V}\end{bmatrix}$$

After performing another transformation $[\mathbf{E},\mathbf{H},\mathbf{V}]^T \rightarrow \left[\mathbf{E},\mathbf{H},\omega_\mathrm{p}^{-1}\mathbf{V}\right]^T$, this Hamiltonian could be reformulated to be hermitian(Equation(1) in main text).

In realistic cold plasma, presence of ions could also be formulated into the Hamiltonian, whose Hermitian form is:

$$\begin{bmatrix} 0 & -c\mathbf{K}\times & -i\omega_{pe}I & -i\omega_{pi}I \\ c\mathbf{K}\times & 0 & 0 & 0 \\ i\omega_{pe}I & 0 & \omega_{ce}\Delta & 0 \\ i\omega_{pi}I & 0 & 0 & -\omega_{ci}\Delta \end{bmatrix}\begin{bmatrix}\mathbf{E}\\ \mathbf{H}\\ \mathbf{V}_e\\ \mathbf{V}_i\end{bmatrix} = \omega\begin{bmatrix}\mathbf{E}\\ \mathbf{H}\\ \mathbf{V}_e\\ \mathbf{V}_i\end{bmatrix}$$

Here the sub-indexes e, i stand for electrons and protons respectively.
Take protons for example, band structures with $\omega_\mathrm{ce} = 1.2\omega_\mathrm{pe}$ and exaggerated parameters of proton, $\omega_\mathrm{ci} = 0.25\omega_\mathrm{ce}$is given in Supplementary Figure 1 (a), which is in agreement with supplementary reference 1. In comparison to the case $\omega_\mathrm{cp} = 0$ shown in Supplementary Figure 1(b), we can see introduction of proton merely adds an extra low energy band, and doesn't influence much of the behaviour on high frequencies around ω_p. Realistically, we have $\omega_\mathrm{ci} = \frac{m_\mathrm{e}}{m_\mathrm{p}}\omega_\mathrm{ce} \approx \omega_\mathrm{pe}/1836$, and $\omega_\mathrm{pi} = \sqrt{\frac{m_\mathrm{e}}{m_\mathrm{p}}}\omega_\mathrm{pe} \approx \omega_\mathrm{pe}/43$. Thus the heavy ions could generally be neglected.

Supplementary Note 2. Derivation of infinite-k Hamiltonian and the associated Berry curvatures

To understand the behaviour of the Berry curvature at very large k, we construct a high-k effective Hamiltonian. First, we can separate the original Hamiltonian described by equation (3) in the main text by two parts, as:

$$H_0 = \begin{bmatrix} 0 & -c\mathbf{K}\times & 0 \\ c\mathbf{K}\times & 0 & 0 \\ 0 & 0 & 0 \end{bmatrix} \quad \text{and} \quad H_1 = \begin{bmatrix} 0 & 0 & -i\omega_{\mathrm{p}} \\ 0 & 0 & 0 \\ i\omega_{\mathrm{p}} & 0 & \omega_{\mathrm{c}}\Delta \end{bmatrix}$$

For Hamiltonian H_0, there are five zero energy states read as:

$$|\lambda_1\rangle = \frac{1}{\sqrt{K_z^{\ 2}+K_x^{\ 2}+K_y^{\ 2}}}\begin{bmatrix} K_x \\ K_y \\ K_z \\ 0 \\ 0 \\ 0 \\ 0 \\ 0 \\ 0 \end{bmatrix} |\lambda_2\rangle = \frac{1}{\sqrt{K_z^{\ 2}+K_x^{\ 2}+K_y^{\ 2}}}\begin{bmatrix} 0 \\ 0 \\ 0 \\ K_x \\ K_y \\ K_z \\ 0 \\ 0 \\ 0 \end{bmatrix} |\lambda_3\rangle = \begin{bmatrix} 0 \\ 0 \\ 0 \\ 0 \\ 0 \\ 0 \\ 1 \\ 0 \\ 0 \end{bmatrix} |\lambda_4\rangle = \begin{bmatrix} 0 \\ 0 \\ 0 \\ 0 \\ 0 \\ 0 \\ 0 \\ 1 \\ 0 \end{bmatrix} |\lambda_5\rangle = \begin{bmatrix} 0 \\ 0 \\ 0 \\ 0 \\ 0 \\ 0 \\ 0 \\ 0 \\ 1 \end{bmatrix}$$

The hyperbolic EFSs that have finite eigen-values at infinite momentum interact only within each other through H_1. Thus the interactions with high energy bands could be neglected. Based on this idea, we can diagonalize the Hamiltonian on basis of the zero energy modes that are expected to form hyperbolic EFSs at infinity.

After excluding the trivial eigen-state $|\lambda_2\rangle$ that does not interact with any other eigen-states, we end up with the effective Hamiltonian at infinity in spherical coordinate.

$$H_\infty = \omega_p \begin{bmatrix} 0 & -i\cos(\phi)\sin(\theta) & -i\sin(\phi)\sin(\theta) & -i\cos(\theta) \\ i\cos(\phi)\sin(\theta) & 0 & i\omega_c/\omega_p & 0 \\ i\sin(\phi)\sin(\theta) & -i\omega_c/\omega_p & 0 & 0 \\ i\cos(\theta) & 0 & 0 & 0 \end{bmatrix}$$

On the other hand, the two higher energy bands of Hamiltonian H_0 have eigen-values $\mathrm{K_r} = \pm\sqrt{\mathrm{K}_x^2+\mathrm{K}_y^2+\mathrm{K}_z^2}$. It is straightforward to see that these eigen states does not interact with each other through interaction Hamiltonian H_1. Thus at infinitely large momentum, where interaction between high energy states and low energy states can be neglected, H_1 does not contribute to these high energy states, meaning that the Berry curvatures must be zero.

The above large k Hamiltonian contains only the polar angle θ and the azimuthal angle φ, which greatly simplifies the calculation. Because gauge fluxes are necessarily along radial directions, we can project the Berry curvatures on to a unit sphere, where Berry curvatures can be readily visualized. As is shown in Supplementary Figure 3, the Berry curvature does not vanish, and is a function of ω_c and θ. When $\omega_c \gg \omega_p$, the Berry curvature approaches zero. In Supplementary Figure 3(b,d), we present radial direction's Berry curvatures as function of θ at different ω_c. The integrated Chern number over a solid angle defined by θ is shown in Supplementary Figure 3(c, e). Interestingly, even though integration over the whole unit sphere is strictly zero, it is -1(+1) on the north (south) hemisphere when $\omega_c < \omega_p$, while is 0 when $\omega_c > \omega_p$. This could be understood as a topological transition at infinity at $\omega_c = \omega_p$ where a pair of Weyl point goes to infinity in the momentum space. Since Weyl points are the only source/drain of gauge flux, there should be a generalized Gauss-Bonnet theorem for this system written as:

$$\frac{1}{2\pi}\iint_\Gamma \vec{\Omega}\cdot d\vec{s} = \pm\sum_v \gamma_v + \frac{1}{2\pi}\iint \vec{\Omega}_\infty \cdot \vec{n}\, d\theta\, d\phi \qquad (1)$$

The above equations states that the Chern number on the hyperbolic EFSs are the overall integral of the gauge flux from the 'Weyl points' subtracting those leaking into the infinity (Supplementary Figure 3 a). The

integration range of the second term on the right hand side is the solid angle characterized by asymptote of hyperbolic EFSs. Sign $\pm$ is determined by which band is being calculated (- for lower band, and + for upper band). v is the number of Weyl points enclosed between the Hyperbolic EFS Γ and the infinity, and γ_v is the Chirality of each Weyl point. It follows that when $\vec{\Omega}_\infty$ vanishes, hyperbolic EFS Γ must have quantized Chern number in all frequency range. However, for magnetized plasma, $\vec{\Omega}_\infty$is generally not zero, Chern number on the hyperbolic EFS is only quantized in some extreme parameters. Nevertheless, Supplementary Equation 1 provides a simple scheme to determine Chern number on an open 'Fermi surface', which is simply determined by the number of Weyl points and the Hamiltonian at infinity. We can verify Supplementary Equation 1 by computing both its left hand side and the right hand side. They are found to be perfectly matched as is shown in Supplementary Figure 4,

Method established here is quite general, and could be extended to photonic materials with low symmetry. So far, we have shown that the plasmon 'Weyl points' are the only source of Berry flux in magnetized plasma, however non-vanishing Berry curvature distribution at infinity would influence how much Berry flux is collected by hyperbolic EFSs.

Supplementary Note 3. Proof of symmetry relation $\Omega(-\mathbf{K},\omega)=\Omega(\mathbf{K},\omega)$

Expression of the Berry curvature at momentum **K** is:

$$\mathbf{\Omega}_n(\mathbf{K}) = i\sum_{m\neq n}\frac{\langle n(\mathbf{K})|\partial_{\mathbf{K}}H(\mathbf{K})|m(\mathbf{K})\rangle\times\langle m(\mathbf{K})|\partial_k H(\mathbf{K})|n(\mathbf{K})\rangle}{(\omega_m(\mathbf{K})-\omega_n(\mathbf{K}))^2} \qquad (2)$$

Operator: $T=\begin{bmatrix}1 & & \\ & -1 & \\ & & 1\end{bmatrix}$ is the momentum-reversal operator: $|n(-\mathbf{K})\rangle = T|n(\mathbf{K})\rangle$, more explicitly because:

$$\omega T\begin{bmatrix}\mathbf{E}\\ \mathbf{H}\\ \mathbf{V}\end{bmatrix} = T\begin{bmatrix}0 & -c\mathbf{K}\times & -i\\ c\mathbf{K}\times & 0 & 0\\ i\omega_{\mathrm{p}}^2 & 0 & \omega_{\mathrm{c}}\Delta\end{bmatrix}TT\begin{bmatrix}\mathbf{E}\\ \mathbf{H}\\ \mathbf{V}\end{bmatrix}$$

$$\omega\begin{bmatrix}\mathbf{E}\\ \mathbf{-H}\\ \mathbf{V}\end{bmatrix} = \begin{bmatrix}0 & c\mathbf{K}\times & -i\\ -c\mathbf{K}\times & 0 & 0\\ i\omega_{\mathrm{p}}^2 & 0 & \omega_{\mathrm{c}}\Delta\end{bmatrix}\begin{bmatrix}\mathbf{E}\\ \mathbf{-H}\\ \mathbf{V}\end{bmatrix}$$

Hence the eigen-state of the flipped momentum is linked to the original state through the relationship:

$$|n(-\mathbf{K})\rangle = T|n(\mathbf{K})\rangle \,\&\, H(-\mathbf{K}) = TH(\mathbf{K})T$$

Then Berry curvature in Supplementary Equation 2 is given by:

$$\mathbf{\Omega}_n(\mathbf{K}) = i\sum_{m\neq n}\frac{\langle n(\mathbf{K})|TT\partial_{\mathbf{K}}H(\mathbf{K})TT|m(\mathbf{K})\rangle\times\langle m(\mathbf{K})|TT\partial_{\mathbf{K}}H(\mathbf{K})TT|n(\mathbf{K})\rangle}{(\omega_m(\mathbf{K})-\omega_n(\mathbf{K}))^2}$$
$$= i\sum_{m\neq n}\frac{\langle n(-\mathbf{K})|\partial_k H(-\mathbf{K})|m(-\mathbf{K})\rangle\times\langle m(-\mathbf{K})|\partial_{\mathbf{K}}H(-\mathbf{K})|n(-\mathbf{K})\rangle}{(\omega_m(-\mathbf{K})-\omega_n(-\mathbf{K}))^2} = \mathbf{\Omega}_n(-\mathbf{K})$$

The above equation shows that when ω_c=0 Berry curvatures vanish for all eigen-states.

Supplementary Note 4. $k \cdot p$ theory for magnetized plasma at Weyl point

From the effective Hamiltonian described in Equation (3) in the main text, we can write the Hamiltonian at $\mathbf{K} = \mathbf{K}_0$ (where the Weyl degeneracy takes place) as:

$$H(\mathbf{K}_0)\left|n(\mathbf{K}_0)\right\rangle = \omega_n(\mathbf{K}_0)\left|n(\mathbf{K}_0)\right\rangle$$

Expanding the Hamiltonian around k_0, we obtain:

$$H(\mathbf{K}_0 + \Delta\mathbf{K}) = H(\mathbf{K}_0) + \frac{\partial H}{\partial \mathbf{K}}\Delta\mathbf{K}$$

By multiplying the Hamiltonian from left with a unity operator $\sum_J |\mathrm{J}(\mathbf{K}_0)\rangle\langle \mathrm{J}(\mathbf{K}_0)|$ to the both sides of the above equation, we arrive at:

$$\frac{\partial H}{\partial \mathbf{K}}\Delta\mathbf{K}\sum_J C_{Jn}\left|\mathrm{J}(\mathbf{K}_0)\right\rangle = [\omega_n(\mathbf{K}_0 + \Delta\mathbf{K}) - \omega_J(\mathbf{K}_0)]\sum_J C_{Jn}\left|\mathrm{J}(\mathbf{K}_0)\right\rangle$$

where $C_{Jn} = \langle \mathrm{J}(\mathbf{K}_0)|n(\mathbf{K}_0 + \Delta\mathbf{K})\rangle$. it follows that

$$\sum_J [\left\langle \mathrm{I}(\mathbf{K}_0)\right|\frac{\partial H}{\partial \mathbf{K}}\Delta\mathbf{K}\left|\mathrm{J}(\mathbf{K}_0)\right\rangle + \omega_J(\mathbf{K}_0)\delta_{IJ}]C_{Jn} = \sum_J \omega_n(\mathbf{K}_0 + \Delta\mathbf{K})\delta_{IJ}C_{Jn}$$

It can be further expressed as

$$\hat{H}C = EC$$

$$\hat{H} = [\left\langle \mathrm{I}(\mathbf{K}_0)\right|\frac{\partial H}{\partial \mathbf{K}}\Delta\mathbf{K}\left|\mathrm{J}(\mathbf{K}_0)\right\rangle + \omega_J(\mathbf{K}_0)\delta_{IJ}]$$

$$E = \omega_n(\mathbf{K}_0 + \Delta\mathbf{K})$$

Till now, the expression of the Hamiltonian is exact. At the Weyl degeneracy, we only need to focus on the interaction between the two states with two-fold degeneracy, and treat the interactions with other states into high order terms. To accomplish this, we can re-phrase the Hamiltonian as

$$\begin{bmatrix} H^{AA} & H^{AB} \\ H^{BA} & H^{BB} \end{bmatrix}\begin{bmatrix} C^A \\ C^B \end{bmatrix} = E\begin{bmatrix} C^A \\ C^B \end{bmatrix}$$

$$H^{AA}C^A - H^{AB}(H^{BB} - \mathrm{I}E)^{-1}H^{BA}C^A = EC^A$$

Where A is a submatrix containing the two degenerate eigen-state at Weyl degeneracy, and B contains all other states, then

$$H^{AA} = [\left\langle I(\mathbf{K}_0)\right|\frac{\partial H}{\partial \mathbf{K}}\Delta\mathbf{K}\left|J(\mathbf{K}_0)\right\rangle + \omega_J(\mathbf{K}_0)\delta_{IJ}]$$

$$H^{AB}(H^{BB} - \mathrm{I}E)^{-1}H^{BA} = \sum_{L\in B}\frac{\left\langle I(\mathbf{K}_0)\right|\frac{\partial H}{\partial \mathbf{K}}\Delta\mathbf{K}\left|K(\mathbf{K}_0)\right\rangle\left\langle K(\mathbf{K}_0)\right|\frac{\partial H}{\partial \mathbf{K}}\Delta\mathbf{K}\left|J(\mathbf{K}_0)\right\rangle}{E_L(\mathbf{K}_0) - E_a}$$

$$I, J \in A$$

$$L \in B$$

By substituting the following expressions for the eigen states into the above equations (gauge of the eigen states wouldn't affect Weyl point's chirality).

$$\left|I\right\rangle = \frac{1}{\sqrt{2(\varepsilon_{12}{}^2 - \varepsilon_{12} + 2)}}\begin{bmatrix} 1 & i & 0 & -i\sqrt{\varepsilon_{12}} & \sqrt{\varepsilon_{12}} & 0 & i(\varepsilon_{12} - 1) & -(\varepsilon_{12} - 1) & 0 \end{bmatrix}^T$$

$$\left|J\right\rangle = \begin{bmatrix} 0 & 0 & \frac{\sqrt{2}}{2} & 0 & 0 & 0 & 0 & 0 & \frac{\sqrt{2}}{2}i \end{bmatrix}^T \qquad (3)$$

where $\varepsilon_{12} = \varepsilon_1 + \varepsilon_2 = \omega_c/(\omega_c - \omega_p)$, and ε_1 and ε_2 are diagonal and gyroelectric terms in the permittivity tensor.

$$\hat{\varepsilon} = \begin{bmatrix} \varepsilon_1 & -i\varepsilon_2 & \\ i\varepsilon_2 & \varepsilon_1 & \\ & & 0 \end{bmatrix}$$

We have the effective Hamiltonian of the outer Weyl point in positive direction:

$$H_1 = \begin{bmatrix} Mk_z & N_x k_x - iN_y k_y \\ N_x k_x + iN_y k_y & 0 \end{bmatrix}$$

$$M = \frac{\sqrt{\varepsilon_{12}}}{\varepsilon_{12}{}^2 - \varepsilon_{12} + 2}$$

$$N_x = -\frac{\sqrt{\varepsilon_{12}}}{2\sqrt{\varepsilon_{12}{}^2 - \varepsilon_{12} + 2}}$$

$$N_y = -\frac{\sqrt{\varepsilon_{12}}}{2\sqrt{\varepsilon_{12}{}^2 - \varepsilon_{12} + 2}}$$

Where the coordinate origin is shifted to the Weyl point and $k_{x,y,z}$ represents small deviations from the degeneracy in momentum space. Then it can be expressed concisely as,

$$H = N_x k_x \sigma_x + N_y k_y \sigma_y \frac{M}{2} k_z \sigma_z + \frac{M}{2} k_z I \qquad (4)$$

When $\omega_c = 1.2$, we have field components at the outer Weyl degeneracy: $M = \frac{\sqrt{6}}{16}$, $N_{x,y} = \frac{\sqrt{3}}{8}$ 。

Generally, $M, N_{x,y}$ could be summarized as:

$$M = \frac{\sqrt{\varepsilon_{12}}}{\varepsilon_{12}{}^2 - \varepsilon_{12} + 2} \frac{1}{\omega_c} \mathbf{S} \cdot \mathbf{B}_s$$

$$N_x = \frac{\sqrt{\varepsilon_{12}}}{2\sqrt{\varepsilon_{12}{}^2 - \varepsilon_{12} + 2}}$$

$$N_y = \frac{\sqrt{\varepsilon_{12}}}{2\sqrt{\varepsilon_{12}{}^2 - \varepsilon_{12} + 2}} \frac{1}{\omega_c} \boldsymbol{\sigma}_s \cdot \mathbf{B}_s$$

For all the Weyl points in magnetized plasma. Here **S** is an unit vector along the Poynting vector of the helical propagating mode , and $\boldsymbol{\sigma}_s$ is the spin vector of the helical propagating mode defined by $\boldsymbol{\sigma}_s = 2(\mathrm{Re}\mathbf{E} \times \mathrm{Im}\mathbf{E})/|\mathbf{E}|^2$.

We could further include interactions with other eigen-states, and get the effective Hamiltonian to the second order, which is:

$$H = \mathbf{d}.\boldsymbol{\sigma} + \varepsilon(\mathbf{k})\mathbf{I}$$

$$\mathbf{d} = [\mathrm{d}_1, \mathrm{d}_2, \mathrm{d}_3]$$

$$d_1 = (Dk_z + N)k_x$$

$$d_2 = -(Dk_z + N)k_y$$

$$d_3 = \frac{1}{2}Mk_z + \frac{1}{2}Ck_z{}^2 + \frac{1}{2}(B - A)(k_x{}^2 + k_y{}^2)$$

$$\varepsilon(\mathbf{k}) = 1 + \frac{1}{2}Mk_z + \frac{1}{2}Ck_z{}^2 + \frac{1}{2}(B + A)(k_x{}^2 + k_y{}^2)$$

Note this Hamiltonian is similar to that of a single spin in the Quantum spin Hall Effect, where the momentum k_z plays the role of the thickness of the Quantum Well, with a topological transition at $(Mk_z + Ck_z{}^2)/(B - A) = 0$

Supplementary Note 5. Chern number of the parabolic 'Fermi surface' of plasmon 'Weyl point'

From the properties of Pauli matrices, we can readily obtain the analytical expression of the Berry curvatures from the plasmon 'Weyl points' (Supplementary Equation 4)as:

$$\boldsymbol{\Omega}(\lambda_{\mathrm{down}}) = -\boldsymbol{\Omega}(\lambda_{\mathrm{up}}) = \gamma \frac{\alpha}{2\left(\alpha^2 k_z^2 + k_r^2\right)^{3/2}}\mathbf{k}$$

This is can be integrated over the parabolic 'Fermi surface', a 2-D manifold expressed as

$$k_z = -\frac{\omega^2 - N^2 k_x{}^2 - N^2 k_y{}^2}{\omega M}$$

This parabolic 2-D manifold could be parameterized as:

$$X(\mathrm{x}, \mathrm{y}, \mathrm{z}) = (\mathrm{x}, \mathrm{y}, -\frac{\omega^2 - N^2 x^2 - N^2 y^2}{\omega M})$$

$$\frac{\partial X}{\partial x} = (1, 0, \frac{2N^2 x}{\omega M})$$

$$\frac{\partial X}{\partial y} = (0, 1, \frac{2N^2 y}{\omega M})$$

and the integration is expressed as

$$\iint_{x,y} \vec{\Omega} \cdot (\frac{\partial X}{\partial x} \times \frac{\partial X}{\partial y}) dxdy$$

Due to rotation symmetry of this system, the above integration is expressed as:

$$-4\pi\gamma \int_r \frac{\omega^2 N^2 r}{(\omega^2 + N^2 r^2)^2} dr = \gamma \left. \frac{2\pi\omega^2}{(\omega^2 + N^2 r^2)} \right|_0^\infty = -2\pi\gamma$$

This leads to a quantized Chern number is of -1.

Supplementary Note 6. Effect of loss on Weyl points

When loss is introduced in the magnetized plasma, Hamiltonian described by equation (1) in the main text becomes a non-hermitian matric:

$$H = \begin{bmatrix} 0 & -c\mathbf{K}\times & -i \\ c\mathbf{K}\times & 0 & 0 \\ i\omega_{\mathrm{p}}{}^{2} & 0 & \omega_{\mathrm{c}}\Delta - i\Gamma I \end{bmatrix}$$

Here Γ is the collision coefficient introduced in equation (1) in main text, I is unity matric. Then we need to look at both the real and imaginary part of its eigenvalues (frequency ω). The longitudinal plasma mode, whose dispersion is a straight line in momentum space in absence of loss (Figure. 1a in main text), become straight lines in both real and imaginary part of eigen values that reads:

$$\omega_{\mathrm{p}}\sqrt{1-\frac{\Gamma^{2}}{4\omega_{\mathrm{p}}{}^{2}}} - i\frac{\Gamma}{2}$$

Further, Weyl point's effective Hamiltonian becomes:

$$H = -i\frac{\Gamma}{2} + \begin{bmatrix} \alpha k_{\mathrm{z}} - i\beta & v_{\mathrm{x}}k_{\mathrm{x}} - iv_{\mathrm{y}}k_{\mathrm{y}} \\ v_{\mathrm{x}}k_{\mathrm{x}} + iv_{\mathrm{y}}k_{\mathrm{y}} & 0 \end{bmatrix}$$

Where β is the difference of eigen value's imaginary part between the longituinal mode and the helical propagating mode, α is a complex number. Band structures of this effective Hamiltonian in $k_x(k_y) - k_z$ plane with $\Gamma = 0.1\omega_{\mathrm{p}}$ are shown in Supplementary Figure 7. The former Weyl point degeneracy reduces to two exceptional points (thus exceptional ring in $k_{\mathrm{x}} - k_{\mathrm{y}}$ plane) residing at $k_{\mathrm{x}} = \pm\frac{\beta}{2}, k_{\mathrm{z}} = 0$. The real parts are in degeneracy when $|k_x| < \frac{\alpha}{2}, k_z = 0$, and imaginary part are in degeneracy when $|k_x| > \frac{\alpha}{2}, k_z = 0$. It has been shown in previous literature [1] that the two exceptional points together gives the same Berry phase as a singular degeneracy when β=0; Thus, even though loss split one Weyl point into two exceptional points, the topological structure stays the same.

Supplementary Note 7. Analytical expression of Berry curvatures at the origin of momentum space:

From the standard $k \cdot p$ theory in Supplementary Note 5, we can readily deduce the effective Hamiltonian at the origin as:

$$i\omega_{c}K_{z}\begin{bmatrix} 0 & -K_{z} & K_{y} \\ K_{z} & 0 & -K_{x} \\ -K_{y} & K_{x} & 0 \end{bmatrix}$$

For ease of derivation, the above equation is transformed into the spherical coordinate as

$$\begin{bmatrix} 0 & -i\cos(\theta) & i\sin(\theta)\sin(\phi) \\ i\cos(\theta) & 0 & -i\sin(\theta)\cos(\phi) \\ -i\sin(\theta)\sin(\phi) & i\sin(\theta)\cos(\phi) & 0 \end{bmatrix}$$

Next we can derive the eigen-states under spherical coordinate as:

$$|\lambda_1\rangle = \frac{\sqrt{2}}{2}\begin{bmatrix} -\cos(\phi)\cos(\theta) - \mathrm{i}\sin(\phi) \\ -\sin(\phi)\cos(\theta) + \mathrm{i}\cos(\phi) \\ \sin(\theta) \end{bmatrix} \quad |\lambda_2\rangle = \frac{\sqrt{2}}{2}\begin{bmatrix} -\cos(\phi)\cos(\theta) + \mathrm{i}\sin(\phi) \\ -\sin(\phi)\cos(\theta) - \mathrm{i}\cos(\phi) \\ \sin(\theta) \end{bmatrix} \quad |\lambda_3\rangle = \begin{bmatrix} \cos(\phi)\sin(\theta) \\ \sin(\phi)\sin(\theta) \\ \cos(\theta) \end{bmatrix}$$

Where $\lambda_1 = 1, \lambda_2 = -1, \lambda_3 = 0$ are the eigen-values. It follows that the Berry curvature of $|\lambda_1\rangle$ is given by:

$$\Omega_{\theta\phi} = -\sin(\theta)$$

Note that this expression is only valid when $K_z > 0$. When $K_z < 0$, since the presence of K_z in the original Hamiltonian, eigen states are changed to:

$$|\lambda_1\rangle = \frac{\sqrt{2}}{2}\begin{bmatrix} -\cos(\phi)\cos(\theta)+\mathrm{i}\sin(\phi) \\ -\sin(\phi)\cos(\theta)-\mathrm{i}\cos(\phi) \\ \sin(\theta) \end{bmatrix} |\lambda_2\rangle = \frac{\sqrt{2}}{2}\begin{bmatrix} -\cos(\phi)\cos(\theta)-\mathrm{i}\sin(\phi) \\ -\sin(\phi)\cos(\theta)+\mathrm{i}\cos(\phi) \\ \sin(\theta) \end{bmatrix} |\lambda_3\rangle = \begin{bmatrix} \cos(\phi)\sin(\theta) \\ \sin(\phi)\sin(\theta) \\ \cos(\theta) \end{bmatrix}$$

And the Berry curvature of $|\lambda_1\rangle$ is

$$\Omega_{\theta\phi} = \sin(\theta)$$

The Berry curvatures in the Cartesian coordinate can be derived from the Jacobian:

$$\Omega_{R_1R_2} = \Omega_{\theta\phi}\frac{\partial(R_1, R_2)}{\partial(\theta,\phi)}$$

The final expression is given by:

$$\begin{cases} \mathbf{\Omega_k} = -\dfrac{\mathbf{K}}{|\mathbf{K}|^3}(K_z>0) \\ \mathbf{\Omega_k} = \dfrac{\mathbf{K}}{|\mathbf{K}|^3}(K_z<0) \end{cases}$$

Supplementary Note 8. Polarization structures of eigen-fields around the plasmon 'Weyl point'

At $\omega = \omega_p$ where plasmon 'Weyl points' are present, magnetized plasma can be separated into two distinct optical modes, whose 'Fermi surfaces' are expressed by:

$$k_x^2 + k_y^2 = 0 \quad \text{and} \quad k_x^2 + k_y^2 + k_z^2 = 1$$

Eigen-states of the 'Weyl points' are included in the first optical mode. The second equation describes a unit sphere, whose polarization state will be discussed later (it will be referred to as the 'background mode' later in the text). For the plasmon Weyl point mode with a nonzero k_x, the wave is decaying along y direction into the magnetized plasma with k_y of the plasmon 'Weyl point' mode is expressed as $k_y = i|k_x|$. Optical mode with this decaying momentum can be solved by substituting k_y into the effective Hamiltonian (Supplementary equation 2):

Thus for positive k_x and negative k_x we have two different characteristic matrixes:

$$\begin{bmatrix} Mk_z & 2N_x k_x \\ 0 & 0 \end{bmatrix} \quad \text{and} \quad \begin{bmatrix} Mk_z & 0 \\ 2N_x k_x & 0 \end{bmatrix}$$

The nonzero solutions to the above effective Hamiltonian are:

$$|k_x > 0\rangle = \begin{bmatrix} k_x \\ -\alpha k_z \end{bmatrix} \quad \text{and} \quad |k_x < 0\rangle = \begin{bmatrix} 0 \\ 1 \end{bmatrix}$$

The basis of the above vectors, i.e. the eigen fields right at the plasmon 'Weyl points' , are expressed by Supplementary Equations 3 :

At the boundary, we are only concerned with the in-plane electric and magnetic fields. These components of the eigen-fields of the evanescent mode with nonzero k_x are given as

$$|k_x>0\rangle = \begin{bmatrix} k_x E_x \\ -\alpha k_z E_z \\ k_x H_x \\ 0 \end{bmatrix} = \frac{1}{\sqrt{2(\varepsilon_{12}{}^2-\varepsilon_{12}+2)}} \begin{bmatrix} k_x \\ 2k_z \\ -i\sqrt{\frac{\omega_c}{\omega_c-\omega_p}}k_x \\ 0 \end{bmatrix}$$

$$|k_x<0\rangle = \frac{1}{\sqrt{2(\varepsilon_{12}{}^2-\varepsilon_{12}+2)}} \begin{bmatrix} 0 \\ 2k_z \\ 0 \\ 0 \end{bmatrix}$$

Suppose eigen-fields resulted from the background mode is expressed by $[E_x' \;\; E_z' \;\; H_x' \;\; H_z']$, then under the PEC boundary condition slope approaching the plasmon 'Weyl point' is:

$$\frac{k_z}{k_x} = \frac{E_x E_z'}{-\alpha E_z E_x'}$$

The eigen field of the back ground mode can be solved as:

$$|\lambda_{background}\rangle = \begin{bmatrix} \sqrt{\frac{\omega_c-\omega_p}{\omega_p}} & -i\frac{\omega_p}{\omega_c}\sqrt{\frac{\omega_c-\omega_p}{\omega_p}} & -\sqrt{\frac{\omega_c-\omega_p}{\omega_c}} & 0 & \sqrt{\frac{\omega_c}{\omega_p}} & -i \end{bmatrix}^T$$

The polarization of the background mode is calculated by the full Hamiltonian and its projection to the xz plane (interface) is plotted as in Supplementary Figure 8.

Thus the slope of the 'Fermi arc' approaching the 'Wey point' when it is interfaced with PEC is given by:

$$\frac{k_z}{k_x} = \frac{E_x}{\alpha E_z}\sqrt{\frac{\varepsilon_2}{\varepsilon_1}} = -\frac{1}{2}\sqrt{\frac{\varepsilon_2}{\varepsilon_1}} = -\frac{1}{2}\sqrt{\frac{\omega_p}{\omega_c}}$$

Supplementary Note 9. Polarization eigen-states around the Weyl point

The polarization eigen-states around the Weyl point could be detected by a spherical prism configuration shown in Supplementary Figure 9.Weyl point is marked by the red spot.

When h=0, based on the eigen fields around Weyl point described in part 7, reflection near Weyl point could be formulated as:

$$\begin{bmatrix} 1 & \sqrt{\frac{\omega_c}{\omega_p}} & 0 & 1 \\ 2\frac{k_z}{k_x} & -1 & -\cos\theta & 0 \\ -i\sqrt{\varepsilon_c}\sin\theta & 0 & \sqrt{\varepsilon_c} & 0 \\ 0 & -i\sqrt{\varepsilon_c}\sin\theta & 0 & \sqrt{\varepsilon_c}\cos\theta \end{bmatrix} \begin{bmatrix} a \\ b \\ \cos\zeta \\ e^{i\zeta}\sin\zeta \end{bmatrix} = \lambda \begin{bmatrix} 0 & 1 \\ \cos\theta & 0 \\ \sqrt{\varepsilon_c} & 0 \\ 0 & -\sqrt{\varepsilon_c}\cos\theta \end{bmatrix} \begin{bmatrix} \cos\zeta \\ e^{i\zeta}\sin\zeta \end{bmatrix}$$

Where $\theta = \arcsin\sqrt{\frac{\omega_c}{\varepsilon_c(\omega_c-\omega_p)}}$ is the incident angle from high refractive index medium to the Weyl point. While $k_{x,z}$ are real, it is possible to show that $e^{i\zeta}$is imaginary, meaning the orientation angle of eigen-states are all parallel to their local coordinates. Here we let $e^{i\zeta} = i$. Solving the matric, and define$\frac{k_z}{k_x} = \xi$,we have:

$$\xi = -\sqrt{\frac{\omega_p}{\omega_c}}\frac{1}{2}\cos^2\theta - \sqrt{\frac{\omega_p}{\omega_c}}\sin\theta\cot 2\zeta$$

$\zeta = \pm\frac{\pi}{4}$ represents circular polarizations, then Eq.(6) in the main text is attained.

Supplementary Note 10. Detection of Chiralities of the Weyl points

The exotic feature of half k-space chirality can be used to detect chirality of the Weyl point. For positive Weyl points, the varying polarization features occur only in the $k_x > 0$ plane. Its evanescent eigen-states can be described by:

$$|k_x > 0\rangle = \begin{bmatrix} k_x \\ -\alpha k_z \end{bmatrix} \qquad |k_x < 0\rangle = \begin{bmatrix} 0 \\ 1 \end{bmatrix}$$

On the contrary, the polarization feature would happen in $k_x < 0$ plane for negative Weyl points. As can be seen from its evanescent eigen-states (given in Supplementary Figure 10)

$$|k_x > 0\rangle = \begin{bmatrix} 0 \\ 1 \end{bmatrix} \qquad |k_x < 0\rangle = \begin{bmatrix} k_x \\ -\alpha k_z \end{bmatrix}$$

With the same set-up as in Fig. 3 (a) of the main text, but with the direction of bias magnetic field reversed (at positive k_z, outer Weyl point's chirality $\gamma = -1$), the half k-space chirality would happen in the left half space as is shown in the following pictures, which are mirror reflection of the ones shown in Fig. 4(a, b) in main text about the y-z plane.

The chirality of the Weyl point is also reflected by the phase distribution of the reflection around the Weyl point, as is shown in Supplementary Figure 11 for both positive (b) and negative (a) Weyl points. Consistent with the Mobius topology of the polarization states in Fig. 4d in the main text, the eigen state rotates two turns to reach the original point. As can be seen from the figure, the phase distributions of the eigen polarization states for the two opposite Weyl points exhibit opposite phase vortices. This therefore is another indication of the chiralities of the Weyl points.

This spiralling phase around the Weyl point will result in a vortex feature of the reflected beam when a TM-polarized Gaussian beam centred on Weyl points is incident onto the magnetized plasma(beam waist is 20 times of free space wavelength to ensure only the momentums near to Weyl point are included). The topological charge of the vortex is consistent with the chirality of the Weyl points as shown in Supplementary Figure 12:

Furthermore, the chirality of the Weyl point is also indicated by the spin texture of the Fermi arc, which is plotted in Supplementary Figure 13. The spin state along the Fermi arc is calculated by using the equation $\boldsymbol{\sigma}_s = 2(\mathrm{Re}\mathbf{E} \times \mathrm{Im}\mathbf{E})/|\mathbf{E}|^2$ (Bliokh, Spin-orbit interactions of light. Nature Photonics **9**, 796-808 (2015).) and is found to be completely in-plane. In the figure, colour of the arrows represents the magnitude of the spin. The chirality can be deduced by the sign of $\mathbf{k}\cdot\boldsymbol{\sigma}_s$ close to the Weyl points.

Supplementary References

1. Kip S. Thorne & Roger D. Blandford. Modern Classical Physics: Optics, Fluids, Plasmas, Elasticity, Relativity, and Statistical Physics. Princeton University Press. ISBN: 9780691159027 (2016)

2. Keck, F., Korsch, H. J. & Mossmann, S. Unfolding a diabolic point: a generalized crossing scenario. *Journal of Physics a-Mathematical and General* . **36**, 2125-2137 (2003).
